# Fructan content in most commonly consumed Slovenian foods and estimation of daily fructan consumption

**DOI:** 10.3389/fnut.2024.1446771

**Published:** 2024-08-02

**Authors:** Blaž Ferjančič, Mojca Korošec, Ana Kočevar Baloh, Jasna Bertoncelj

**Affiliations:** Biotechnical Faculty, University of Ljubljana, Ljubljana, Slovenia

**Keywords:** FODMAPs, irritable bowel syndrome, fructan, daily intake, AOAC 999.03

## Abstract

Fructans can be considered as part of the group of fermentable oligo-, di- and monosaccharides and polyols (FODMAPs). Short-chain fructans have a rapid fermentation rate and can thus worsen symptoms in patients with irritable bowel syndrome. In this study, the fructan content in 40 of the most commonly consumed foods in Slovenia was measured. Overall, the fructan content was relatively low: 0.1–1.97 g/100 g fresh weight. The highest fructan content was found in onion (1.97 g/100 g), followed by wheat flour (0.75 g/100 g). A simple model for calculating fructan intake was developed based on the results of the SI. Menu 2017/2018 national survey, which collected data on the frequency of intake of different foods and food groups (expressed in g/day). After calculating the daily intake of the surveyed foods, we used our results on fructan content to estimate daily fructan consumption. Mean daily fructan intake reaches 1.6–1.7 g/day, with no differences between men and women. Our assessment of fructan intake at the national level represents the first step toward the creation of a database on FODMAP intake.

## 1 Introduction

Fructans are widespread throughout the plant kingdom and are found in monocotyledonous and dicotyledonous plants and green algae. Fructans are linear or branched polymers in which one or more of the β-fructofuranosyl-fructose linkages, such as β (2-1) and β (2-6), constitute the predominant unit. This results in fructooligosaccharides of different sizes (1), ranging from 15 to 200 monomers (2). The fermentation rate of fructans depends on the degree of polymerization, with shorter chains fermenting faster than longer ones (3). In addition to size, fructans can also be classified according to the predominantly present glycosidic bond as either β-2,1-linked inulins or β-2,6-linked levans (4). In this article, the term “fructan” is used for both groups.

Due to their chemical composition and physical functions, fructans belong to the group of dietary fibers (5). Fructans positively affect human health, with health benefits such as lowering serum triglyceride and cholesterol levels, increasing calcium absorption, regulating glucose homeostasis, and relieving constipation and other benefits associated with fermentable dietary fiber (1). Nevertheless, the fermentation of fructans with shorter chains (≤10 monomers) can also cause certain problems. Fructans belong to fermentable oligo-, di- and monosaccharides and polyols (FODMAPs), which rapidly ferment not only in the large intestine but also in the distal part of the small intestine, causing abdominal pain, flatulence, cramps, and altered bowel habits when consumed in higher amounts (6). Skodje et al. (7) demonstrated that fructans are more likely to trigger symptoms in non-celiac gluten-sensitive individuals than gluten itself. However, the exact role of fructans in the diets of patients with gastrointestinal disorders remains to be determined (7). On the one hand, fructans can exacerbate symptoms in patients with irritable bowel syndrome (IBS) because of their rapid fermentation rate. On the other hand, fructans are prebiotics and promote probiotic bacteria, which have a positive effect on colon health (8).

Data on fructan content in foods are important for informed dietary intervention for IBS patients who require low FODMAP intake (9). Because of the different physiological functions of fermentable dietary fiber, which includes fructans, it is important to be able to estimate their dietary intake. Fructans have been shown to modulate human microbiota and promote butyrate production when fermented in the colon (10). Nevertheless, studies on fructan consumption are scarce and mainly limited to IBS patients. Algera et al. (11) reported that mean daily fructan intake is 2.5 g/day (range: 0.7–7.0 g/day), and Liljebo et al. (12) reported that mean daily fructan intake is 3.5 g/day in the Swedish population.

The aim of our study was to estimate the fructan content in food samples with determined low-molecular-weight soluble dietary fiber to obtain more comprehensive data on dietary fiber components in Slovenian foods and to extend our work on dietary fiber determination (13). Using the data obtained, we developed a simple model to estimate fructan intake in the adult Slovenian population as this was never done previously.

## 2 Materials and methods

### 2.1 Samples

The included food samples were selected based on previous data from the Slovenian national food consumption study SI. Menu 2017/2018 (14), which was conducted as part of the international EU.Menu study. In 2022, Ferjančič et al. (13) determined total dietary fiber, including low-molecular-weight soluble dietary fiber, using the AOAC 2011.25 method. In this current study, we excluded liquid samples. Samples (Table 1) were purchased from grocery stores (including supermarkets) or local markets in Slovenia. Composite samples were made from at least three subsamples of different brands of the same food if the food was branded and packaged (e.g., canned beans were an equal mass mixture of red, brown, and white beans). The same principle was applied across different cultivars of vegetables and fruits, with at least three subsamples of these unbranded foods included in composite samples, which contained only edible parts. The samples were cooked, baked, or fried and then dried and weight loss was recorded (at 50 °C overnight; Stermatic ST-11, Zagreb, Croatia). Water content were calculated. Dried samples were ground to a particle size of <0.5 mm. Samples containing >10% fat according to the Slovenian Food Composition Database (15) were defatted according to AOAC 985.29 by treatment with petroleum ether before fructan determination.

**Table 1:** Fructan content in food.

**Sample**	**Water content (%)**	**% FRU (DW)**	**% FRU (FW)**	**% FRU/portion (g)**	**LMWSDF (g/100 g FW)**
**Vegetables**					
Onion	88	16.41 ± 0.40	1.97 ± 0.05	1.38 (70)	4.09 ± 0.70
Carrot	88	0.90 ± 0.11	0.11 ± 0.01	0.12 (110)	1.62 ± 0.43
Kohlrabi	91	ND	ND	ND	0.18 ± 0.10
Tomato	95	0.19 ± 0.03	0.01 ± 0.00	0.01 (148)	0.27 ± 0.03
Lettuce	98	0.61 ± 0.04	0.01 ± 0.00	0.00 (36)	0.03 ± 0.00
Bell pepper	90	0.17 ± 0.04	0.02 ± 0.00	0.01 (119)	0.18 ± 0.06
Cauliflower	89	0.49 ± 0.10	0.09 ± 0.01	0.11 (120)	0.21 ± 0.12
Broccoli	87	0.12 ± 0.02	0.02 ± 0.00	0.03 (148)	1.03 ± 0.46
Cucumber	95	ND	ND	ND	0.14 ± 0.03
Cabbage	91	0.36 ± 0.06	0.03 ± 0.01	0.02 (80)	0.94 ± 0.93
Leek	92	2.96 ± 0.06	0.24 ± 0.00	0.21 (89)	1.57 ± 0.07
Courgette	95	0.16 ± 0.07	0.01 ± 0.00	0.01 (90)	0.64 ± 0.10
Pickle	94	ND	ND	ND	0.15 ± 0.08
Sour turnip	92	ND	ND	ND	4.70 ± 0.29
Sauerkraut	92	ND	ND	ND	2.70 ± 0.33
**Potato**					
Cooked potato	84	ND	ND	ND	0.24 ± 0.02
Baked potato	/	ND	ND	ND	1.75 ± 0.75
French fries	85^*^	ND	ND	ND	1.79 ± 0.55
Crisps	69^*^	ND	ND	ND	2.95 ± 0.13
**Fruits**					
Banana	74	0.42 ± 0.04	0.11 ± 0.01	0.14 (126)	0.76 ± 0.16
Apple	85	0.87 ± 0.04	0.09 ± 0.01	0.22 (242)	2.65 ± 0.20
Orange	77	0.90 ± 0.13	0.21 ± 0.03	0.28 (131)	1.28 ± 0.33
Grape	81	0.33 ± 0.08	0.06 ± 0.01	0.08 (126)	0.83 ± 0.13
Pear	77	0.58 ± 0.07	0.13 ± 0.02	0.22 (166)	0.09 ± 0.03
Tangerine	88	0.76 ± 0.06	0.11 ± 0.01	0.12 (109)	1.80 ± 0.18
**Grains and grain products**					
White bread	38	0.22 ± 0.05	0.14 ± 0.03	0.04 (25)	3.81 ± 0.58
Brown bread	44	0.25 ± 0.04	0.14 ± 0.02	0.04 (29)	6.71 ± 1.10
Wheat flour T500	/	0.75 ± 0.04	0.75 ± 0.04	0.94 (125)	6.06 ± 1.62
Rice (cooked)	79	ND	ND	ND	2.65 ± 0.20
Corn flakes	/	ND	ND	ND	1.53 ± 0.03
Rolled oats	/	0.32 ± 0.01	0.32 ± 0.01	0.48 (159)	1.45 ± 0.06
Pasta (cooked)	55	0.73 ± 0.03	0.33 ± 0.01	0.33 (100)	1.91 ± 0.07
Polenta (cooked)	53	0.07 ± 0.03	0.04 ± 0.01	0.07 (170)	1.03 ± 0.29
Dehulled barley	/	0.47 ± 0.15	0.47 ± 0.15	0.47 (100)	1.27 ± 0.05
**Legumes**					
Canned peas	81	1.70 ± 0.16	0.33 ± 0.03	0.26 (79)	5.07 ± 0.23
Green beans	92	0.39 ± 0.14	0.03 ± 0.01	0.05 (177)	1.07 ± 0.31
Canned beans	87	1.85 ± 0.03	0.24 ± 0.00	0.43 (179)	1.48 ± 0.03
**Nuts**					
Almond^*^	48^**^	1.16 ± 0.09	0.60 ± 0.04	0.17 (28)	1.66 ± 0.23
Hazelnut^*^	33^**^	1.09 ± 0.04	0.73 ± 0.03	0.20 (28)	1.30 ± 0.38
Walnut^*^	44^**^	0.66 ± 0.11	0.37 ± 0.06	0.10 (28)	1.35 ± 0.40

DW, dry weight; FW, fresh weight; ND, non-detectable; LMWSDF, low molecular weight soluble dietary fiber; % FRU/portion (g), percentage of fructan per portion, mass of the portion (as defined in OPEN) in brackets.

^*^Water and fat content combined.

^**^Fat content.

### 2.2 Fructan determination

The extraction and determination of fructans has been described previously (16) and is recognized by the AOAC (AOAC 999.03). The samples were first dried and ground to a particle size of ~0.5 mm. These samples (0.1–0.4 g) were then placed into centrifuge tubes (50 ml), and fructans were extracted with hot water (10 min, 100°C). The samples were then cooled to room temperature, and 2 ml aliquots were transferred to microcentrifuge tubes and centrifuged (13,000 rpm, 5 min, Eppendorf Centrifuge 5415D, Germany). The sample aliquots were transferred to test tubes and treated with solutions of sucrase/amylase, alkaline borohydride solution (to remove sucrose, starch, and reducing sugars), and then fructanase (40°C, 30 min). Next, reagent blanks and sample blanks were prepared. The working reagent 4-hydroxybenzoic acid hydrazide was added to all tubes and incubated in boiling water for 6 min. After cooling the samples and sample blanks in cold water, the absorbance was measured at 410 nm. Samples with a difference in absorbance between sample and sample blank of ≤ 0.02 were below the limit of quantification. The results were quantified in comparison to the reference point for the absorbance of 54.5 μg D-fructose. To verify the results, the control samples included in the enzyme kit were tested with each batch of samples. All reagents and enzymes were obtained in Megazyme Fructan Assay kit.

### 2.3 Calculation of fructan intake

A simple model for calculating fructan intake was developed based on the SI. Menu 2017/2018 national survey results, from which data on the frequency (mode) of intake of different foods and the intake of different food groups (expressed in g/day) was obtained. Six different food groups (the most important food groups for dietary fiber intake) were chosen. As we previously selected the foods to be analyzed, we developed a model based on “forced food selection.” First, we selected the food groups that contribute to dietary fiber and fructan intake. Second, we identified the most common foods in the food groups based on mode. Third, we calculated the contribution of each identified food in a food group as the ratio of the mode of the food versus the sum of modes for all foods in the group. With this, we established the contribution of each identified food to total intake with this food group. Fourth, we multiplied the percentage of food contribution to food group intake with the mass of food group intake and fructan content in the food. This yielded fructan intake for each identified food. Last, we summarized fructan intake for each of 392 subjects (182 men and 210 women) from the SI. Menu 2017/2018 study (14). The estimated daily fructan intake was the mean intake of all subjects. The model was designed and calculated with Microsoft Excel.

## 3 Results

### 3.1 Fructan content in foods

We analyzed the fructan content in 40 of the most consumed foods in Slovenia (Table 1). Additionally, we considered data for low-molecular-weight soluble dietary fiber (13) because fructans are part of this dietary fiber subgroup. Overall, the fructan content in our samples was relatively low: 0.1–1.97 g/100 g fresh weight. The highest fructan content was found in onion (1.97 g/100 g), followed by wheat flour (0.75 g/100 g). Fructans were not detectable in any of the samples only in the potato group. No correlation between fructan content and low-molecular-weight soluble dietary fiber was observed.

### 3.2 Fructan intake in the adult Slovenian population

In the second part of our study, we developed a simple model to estimate approximate fructan intake in the adult Slovenian population (aged 18–65 years). The model was evaluated by comparing estimated dietary fiber intake. The decision to use the same model as for dietary fiber estimation, was based on the fact that fructans represent dietary fiber, and the foods included in the model were the same. Data from our previous study were used to evaluate the model (13). A previous model using data obtained by the AOAC 991.43 method estimated a daily intake of dietary fiber for adults of 17.61 ± 7.45 g/day (17). Koroušić et al. (18) reported 19.7 g dietary fiber/day, based on the food consumption data from SI. Menu 2017/2018. Our model shows that fiber intake is underestimated by 10.6%. However, we only assessed 40 different foods, as opposed to Koroušić et al. (18), who used detailed data from the 24-h recall and food propensity questionnaire from the SI. Menu 2017/2018 survey. Based on this comparison, our model, although simple, provides an adequate rough estimate of dietary fiber and fructan intake. Daily fructan intake is presented in Table 2. Our data indicate that the range of daily fructan intake is 0.2–5.5 g/day, and that mean fructan intake reaches 1.6–1.7 g/day, with no significant differences between men and women. The mean contributions of the main sources of fructan were 0.72 g for onions, 0.41 g for white flour (T500), and 0.13 g for dehulled barley, followed by bananas, apples, and pasta, all contributing 0.06 g.

**Table 2:** Daily fructan intake in the adult Slovenian population.

	**All**	**Male**	**Female**
Mean ± SD (g/day)	1.69 ± 0.84	1.69 ± 0.85	1.61 ± 0.89
Minimum (g/day)	0.21	0.29	0.21
Maximum (g/day)	5.54	5.54	5.53

## 4 Discussion

### 4.1 Fructan content in foods

Our results regarding fructan content are mostly lower than those of other studies. For example, for white bread, we determined 0.14 g/100 g, whereas Lockyer and Stanner (19) reported 0.68 g/100 g. Conversely, our results regarding onions, white rice, pasta, and beans are comparable to those of Lockyer and Stanner (19). The data for fruits and vegetables used in the study by Lockyer and Stanner (19) were obtained by Muir et al. (20), who used the Fructan HK Assay Kit. Different methodologies may explain the discrepancies between our results and theirs. Compared to our study, Biesiekierski et al. (21) reported similar fructan levels for processed foods (e.g., pasta and bread) and similar discrepancies between studies. The enzyme-based analytical method for fructan determination requires testing control material, and thus we may conclude that discrepancies between data derive from the biological variability of foods from different geographical origins.

### 4.2 Dietary fructan intake in the adult Slovenian population

Low fructan intake can be associated with insufficient dietary fiber intake. Liljebo et al. (12) used over 1,800 different values of fructan content in various foods and a 4-day food diary and estimated that the fructan intake of the Swedish population is 3.46 g/day. Barrett and Gibson (22) assessed fructan intake in the Australian population. They reported a daily fructan intake of 3 g based on a 7-day food diary and 3.3 g based on a food frequency questionnaire. These values are twice as high as those in our study. Another study reported daily fructan intake at 2.9–3.9 g/day, with lower daily intakes in patients with Chron's disease and higher intakes in healthy controls (23).

The presented model for estimating fructan intake is a very simple model. We would like to emphasize that this model enables a rough preliminary estimation of fructan intake for the Slovenian population. For a more accurate estimation, a larger number of foods should be investigated. Nevertheless, we have demonstrated how to quickly and easily develop a model for estimating food intake that does not require pre-existing food composition databases. The only prerequisite for this approach is access to data regarding the frequency of food consumption and the mean daily intake of selected foods. For the European Union, these data are available on the European Food Safety Authority website (https://www.efsa.europa.eu/en/data-report/food-consumption-data#the-efsa-comprehensive-european-food-consumption-database). For our model, we used raw national data, as the basis for the model was developed before the data for Slovenia were published. To improve the estimation of fructan intake, each respondent should report dietary intake using a food diary, cross-referenced in the interview with a trained interviewer to avoid under-reporting and ensure good-quality data. This should be followed by extensive analytical work to determine fructan content in foods, as there is no existing database containing this information. Overall, this approach, although more precise, would be a huge undertaking. With our work, we have taken the first step to enabling this kind of research.

### 4.3 The significance of knowing fructan content and intake

As fructans belong to the FODMAP group, the assessment of fructan intake at the national level is a first step toward the creation of a database on FODMAP intake (12, 24, 25). According to Mansueto et al. (26), this enables a better dietary approach to resolving the symptoms of IBS patients with low-FODMAP diets. Dugum et al. (27) in their review of the low-FODMAP approach to reducing IBS symptoms mentioned a website with data on FODMAP content in foods, which could help patients make informed choices when buying foods. However, given the differences in fructan content of foods due to geographical origin (see previous chapter), we advocate the development of a national database. Nevertheless, it should be noted that the inclusion of fructans in the FODMAP group is somewhat controversial. According to Halmos and Gibson (28), the term FODMAP is poorly defined, and its broader meaning also includes fructans with a higher degree of polymerization (e.g., inulin with long chains of >23 monomer units), which exert minimal osmotic effects and exhibit slower fermentation rates. Compared to other fructans, inulin is less soluble and more viscous in solutions, which results in slower fermentation rates (29) and less frequent gastrointestinal symptoms (30). In this study, we considered fructan a FODMAP because our methodology only quantifies total fructan content regardless of the degree of polymerization.

Research on FODMAPs is also gaining attention because of the currently accepted approach to alleviating IBS symptoms—the low-FODMAP diet, which eliminates foods containing FODMAPs and reintroduces tolerated foods. Without knowledge of FODMAP dietary intake or content in foods, this approach can lead to a deficiency of nutrients, especially dietary fibers (31). Furthermore, Varney et al. (32) reported that 0.5 g of FODMAPs per meal is the threshold that does not cause reactions in IBS patients. Our study has set the foundation for adding one of the FODMAP constituents (fructans) to the national food composition database, which would improve the tools available to clinical dietitians working with IBS patients.

### 4.4 Strengths and limitations

This study is based on food consumption data of the Slovenian population, and the selected samples represent the most commonly consumed fructan-containing foods. In this way, we analyzed samples in order of importance based on their contribution to fructan intake. A limitation of this approach is that only a small number of samples was analyzed. This was compensated for by using composite samples to ensure representative and robust results. The greatest strength of the fructan intake estimation is its simplicity and the use of analytical data on fructan content. The model was developed using actual consumption data from 392 adults previously sampled by Gregorič et al. (14). We have proposed an approach to improve the precision of our study in the chapter 4.2.

## 5 Conclusions

In this study, we assessed fructan content in 40 different food samples and determined values of 0.1–1.97 g/100 g fresh weight. These results were used to develop a simple model for estimating fructan intake, which was subsequently verified and the model was confirmed as adequate. This study is the first to estimate daily fructan intake for the adult Slovenian population (at 1.7 g/day). Furthermore, our results represent the first step toward the inclusion of data on fructan content in the national food composition database.

## Data availability statement

The raw data supporting the conclusions of this article will be made available by the authors, without undue reservation.

## Ethics statement

The studies involving humans were approved by National Medical Ethics Committee, Ljubljana, Slovenia (KME 53/07/16; Approval No. 0120-337/2016 issued on 19 July 2016). The studies were conducted in accordance with the local legislation and institutional requirements. Written informed consent for participation was not required from the participants or the participants' legal guardians/next of kin in accordance with the national legislation and institutional requirements.

## Author contributions

BF: Conceptualization, Data curation, Formal analysis, Investigation, Methodology, Supervision, Writing – original draft, Writing – review & editing. MK: Conceptualization, Methodology, Writing – review & editing. AK: Data curation, Investigation, Writing – original draft. JB: Supervision, Writing – review & editing.

## References

[1] Verma DK Patel AR Thakur M Singh S Tripathy S Srivastav PP. A review of the composition and toxicology of fructans, and their applications in foods and health. J Food Compos Anal. (2021) 99:103884. 10.1016/j.jfca.2021.103884

[2] Benkeblia N. Fructooligosaccharides and fructans analysis in plants and food crops. J Chromatogr A. (2013) 1313:54–61. 10.1016/j.chroma.2013.08.013 23988013

[3] Hernot DC Boileau TW Bauer LL Middelbos IS Murphy MR Swanson KS. *In vitro* fermentation profiles, gas production rates, and microbiota modulation as affected by certain fructans, galactooligosaccharides, and polydextrose. J Agric Food Chem. (2009) 57:1354–61. 10.1021/jf802484j 19199596

[4] Roberfroid MB Delzenne NM. Dietary fructans. Annu Rev Nutr. (1998) 18:117–43. 10.1146/annurev.nutr.18.1.117 9706221

[5] Stephen AM Champ MMJ Cloran SJ Fleith M Van Lieshout L Mejborn H. Dietary fibre in Europe: current state of knowledge on definitions, sources, recommendations, intakes and relationships to health. Nutr Res Rev. (2017) 30:149–90. 10.1017/S095442241700004X 28676135

[6] Spiller R. How do FODMAPs work? J. Gastroenterol Hepatol. (2017) 32:36–9. 10.1111/jgh.13694 28244663

[7] Skodje GI Sarna VK Minelle IH Rolfsen K Muir JG Gibson PR. Fructan, rather than gluten, induces symptoms in patients with self-reported non-celiac gluten sensitivity. Gastroenterology. (2018) 154:529–39.e2. 10.1053/j.gastro.2017.10.040 29102613

[8] Wilson B Whelan K. Prebiotic inulin-type fructans and galacto-oligosaccharides: definition, specificity, function, and application in gastrointestinal disorders. J Gastroenterol Hepatol. (2017) 32:64–8. 10.1111/jgh.13700 28244671

[9] Whelan K Martin LD Staudacher HM Lomer MCE. The low FODMAP diet in the management of irritable bowel syndrome: an evidence-based review of FODMAP restriction, reintroduction and personalisation in clinical practice. J Hum Nutr Diet. (2018) 31:239–55. 10.1111/jhn.12530 29336079

[10] Van den Abbeele P Gérard P Rabot S Bruneau A El Aidy S Derrien M. Arabinoxylans and inulin differentially modulate the mucosal and luminal gut microbiota and mucin-degradation in humanized rats. Environ Microbiol. (2011) 13:2667–80. 10.1111/j.1462-2920.2011.02533.x 21883787

[11] Algera JP Störsrud S Lindström A Simrén M Törnblom H. Gluten and fructan intake and their associations with gastrointestinal symptoms in irritable bowel syndrome: a food diary study. Clin Nutr. (2021) 40:5365–72. 10.1016/j.clnu.2021.09.002 34560607

[12] Liljebo T Störsrud S Andreasson A. Presence of fermentable oligo-, di-, monosaccharides, and polyols (FODMAPs) in commonly eaten foods : extension of a database to indicate dietary FODMAP content and calculation of intake in the general population from food diary data. BMC Nutr. (2020) 6:47. 10.1186/s40795-020-00374-3 32963797 PMC7499970

[13] Ferjančič B Skrt M Korošec M Bertoncelj J. Comparative analysis of dietary fibre determination by AOAC 991.43 and AOAC 2011.25 for frequently consumed foods in Slovenia. Food Chem. (2022) 397:133753. 10.1016/j.foodchem.2022.133753 35905619

[14] Gregorič M Blaznik U Delfar N Zaletel M Lavtar D Koroušić Seljak B. Slovenian national food consumption survey in adolescents, adults and elderly. EFSA Support Publ. (2019) 28:EN-1729. 10.2903/sp.efsa.2019.EN-1729

[15] OPEN platform for clinical nutrition. Food Lexicon. (2022). Available online at: https://opkp.si/en_GB/lexicon/search (accessed June, 2024).

[16] McCleary BV Murphy A Mugford D Collaborators. Measurement of total fructan in foods by enzymatic/spectrophotometric method: collaborative study. J AOAC Int. (2000) 83:356–64. 10.1093/jaoac/83.2.356 10772173

[17] Ferjančič B. Application of the AOAC 2011.25 method for dietary fibre determination and its impact on estimated dietary intake [PhD Dissetration]. Ljubljana: University of Ljubljana (2021).

[18] Koroušić Seljak B Valenčič E Hristov H Hribar M Lavriša Ž Kušar A. Inadequate intake of dietary fibre in adolescents, adults, and elderlies: results of slovenian representative SI. Menu Study Nutr. (2021) 13:3826. 10.3390/nu13113826 34836083 PMC8619009

[19] Lockyer S Stanner S. Prebiotics–an added benefit of some fibre types. Nutr Bull. (2019) 44:74–91. 10.1111/nbu.12366

[20] Muir JG Shepherd SJ Rosella O Rose M Barrett JS Gibson PR. Fructan and free fructose content of common Australian vegetables and fruit. J Agric Food Chem. (2007) 55:6619–27. 10.1021/jf070623x 17625872

[21] Biesiekierski JR Rosella O Rose R Liels K Barrett JS Shepherd SJ. Quantification of fructans, galacto-oligosaccharides and other short-chain carbohydrates in processed grains and cereals. J Hum Nutr Diet. (2011) 24:154–76. 10.1111/j.1365-277X.2010.01139.x 21332832

[22] Barrett JS Gibson PR. Development and validation of a comprehensive semi-quantitative food frequency questionnaire that includes FODMAP intake and Glycemic Index. J Am Diet Assoc. (2010) 110:1469–76. 10.1016/j.jada.2010.07.011 20869485

[23] Anderson JL Hedin CR Benjamin JL Koutsoumpas A Ng SC Hart AL. Dietary intake of inulin-type fructans in active and inactive Crohn's disease and healthy controls: a case-control study. J Crohn's Colitis. (2015) 9:1024–31. 10.1093/ecco-jcc/jjv136 26221003

[24] Gibson PR Halmos EP So D Yao C Varney JE Muir JG. Diet as a therapeutic tool in chronic gastrointestinal disorders: lessons from the FODMAP journey. J Gastroenterol Hepatol. (2022) 37:644–52. 10.1111/jgh.15772 34994019

[25] Tuck C Bogatyrev LA Costetsou I Gibson P Barrett J Muir J. Fermentable short chain carbohydrate (FODMAP) content of common plant-based foods and processed foods suitable for vegetarian- and vegan-based eating patterns. J Hum Nutr Diet. (2018) 31:422–35. 10.1111/jhn.12546 29473657

[26] Mansueto P Seidita A D'alcamo A Carroccio A. Role of FODMAPs in patients with irritable Bowel syndrome. Nutr Clin Pract. (2015) 30:665–82. 10.1177/0884533615569886 25694210

[27] Dugum M Barco K Garg S. Managing irritable bowel syndrome: the low-FODMAP diet. Cleve Clin J Med. (2016) 83:655–62. 10.3949/ccjm.83a.14159 27618353

[28] Halmos EP Gibson PR. Controversies and reality of the FODMAP diet for patients with irritable bowel syndrome. J Gastroenterol Hepatol. (2019) 34:1134–42. 10.1111/jgh.14650 30945376

[29] Melilli MG Buzzanca C Di Stefano V. Quality characteristics of cereal-based foods enriched with different degree of polymerization inulin: a review. Carbohydr Polym. (2024) 332:121918. 10.1016/j.carbpol.2024.121918 38431396

[30] Rumessen JJ Gudmand-Høyer E. Fructans of chicory: intestinal transport and fermentation of different chain lengths and relation to fructose and sorbitol malabsorption. Am J Clin Nutr. (1998) 68:357–64. 10.1093/ajcn/68.2.357 9701194

[31] Zugasti Murillo A Estremera Arévalo F Petrina Jáuregui E. Diet low in fermentable oligosaccharides, disaccharides, monosaccharides and polyols (FODMAPs) in the treatment of irritable bowel syndrome: indications and design. Endocrinol Nutr. (2016) 63:132–8. 10.1016/j.endoen.2015.10.014 26830853

[32] Varney J Barrett J Scarlata K Catsos P Gibson PR Muir JG. FODMAPs: food composition, defining cutoff values and international application. J Gastroenterol Hepatol. (2017) 32:53–61. 10.1111/jgh.13698 28244665

